# Modulation of the Paracrine Kynurenic System in Bone as a New Regulator of Osteoblastogenesis and Bone Mineral Status in an Animal Model of Chronic Kidney Disease Treated with LP533401

**DOI:** 10.3390/ijms21175979

**Published:** 2020-08-19

**Authors:** Adrian Mor, Krystyna Pawlak, Bartlomiej Kalaska, Tomasz Domaniewski, Beata Sieklucka, Marta Zieminska, Bogdan Cylwik, Dariusz Pawlak

**Affiliations:** 1 Department of Pharmacodynamics, Medical University of Bialystok, Mickiewicza 2C, 15-222 Bialystok, Poland; adrian.mor@outlook.com (A.M.); bartlomiej.kalaska@umb.edu.pl (B.K.); beataznorko@wp.pl (B.S.); 2 Department of Monitored Pharmacotherapy, Medical University of Bialystok, Mickiewicza 2C, 15-222 Bialystok, Poland; krystynapawlak@poczta.onet.pl (K.P.); tdomaniewski@wp.pl (T.D.); marta.zieminska@umb.edu.pl (M.Z.); 3 Department of Paediatric Laboratory Diagnostics, Medical University of Bialystok, Waszyngtona 17, 15-269 Bialystok, Poland; cylwikb@umb.edu.pl

**Keywords:** LP533401, gut-derived serotonin, chronic kidney disease, mineral and bone disorders, tryptophan metabolism, tryptophan 2,3-dioxygenase, kynurenine pathway, kynurenine, uremic toxins

## Abstract

An increase in the peripheral synthesis of serotonin and kynurenine, observed during the chronic kidney disease (CKD) course, is negatively associated with bone health. Serotonin and kynurenine are connected by the common precursor, tryptophan. LP533401 is an inhibitor of peripheral serotonin synthesis. This study aimed to establish if the inhibition of serotonin synthesis by LP533401 may affect the kynurenine pathway activity in bone tissue and its potential consequence with regard to osteogenesis and bone mineral status. Nephrectomized rats were treated with LP533401 at a dose of 30 and 100 mg/kg daily for eight weeks. Tryptophan and kynurenine concentrations were determined, and tryptophan 2,3-dioxygenase (TDO) expression was assessed. We discovered the presence of a TDO-dependent, paracrine kynurenic system in the bone of rats with CKD. Its modulation during LP533401 treatment was associated with impaired bone mineral status. Changes in TDO expression affecting the kynurenine pathway activity were related to the imbalance between peripheral serotonin and 25-hydroxyvitamin D. There were also close associations between the expression of genes participating in osteoblastogenesis and activation of the kynurenine pathway in the bones of LP53301-treated rats. Our results represent the next step in studying the role of tryptophan metabolites in renal osteodystrophy.

## 1. Introduction

Abnormalities in bone metabolism represent the most complex complications accompanying chronic kidney disease (CKD) development [1,2]. The systemic CKD mineral and bone disorders (CKD-MBD) are associated with the disturbances in calcium and phosphorus metabolism, secondary hyperparathyroidism, deficiency of vitamin D, vascular calcification, and bone tissue disorders [2].

In recent decades, serotonin (5-HT) received intensive attention due to its potential role in bone metabolism [3]. However, the issue of 5-HT and bone biology is still controversial, and it is closely dependent on the site of its synthesis; gut-derived 5-HT has unfavorable effects on bone health, while brain-derived 5-HT has an osteoanabolic effect [4,5,6,7]. Our previous study showed that the elevated levels of peripherally synthesized 5-HT may influence the strength and metabolism of a long bone in nephrectomized rats [8]. We also identified a new molecular pathway, via which elevated circulating 5-HT can affect the expression of 5-HT-dependent genes in bone, shifting in forkhead box protein O1 (FOXO1) target genes from a cAMP-responsive element-binding protein (CREB)- to an activating transcription factor 4 (ATF4)-dependent response, resulting in enhanced osteoblast differentiation in this model [9].

Tryptophan (TRP) is the only precursor of 5-HT, and its conversion into 5-HT is initiated by tryptophan hydroxylase (TPH). Two isoforms of this enzyme (TPH-1 and TPH-2) occur. TPH-1 initiates the synthesis of peripheral 5-HT, mainly in the duodenum, while the second isoform, TPH-2, is exclusively expressed in the central nervous system. LP533401 is a small-molecule inhibitor of TPH-1. LP533401 does not penetrate the blood–brain barrier and does not lead to disturbances of the central 5-HT [10]. Yadav et al. also showed that pharmacological inhibition of TPH-1 by LP533401 was able to prevent bone loss in ovariectomized animals [10]. Recently, we demonstrated that LP533401 decreases plasma 5-HT concentration and may improve bone mineralization in nephrectomized rats [11].

Kynurenine (KYN), the major metabolite of TRP, is synthesized in the body by the tryptophan 2,3-dioxygenase (TDO) and indoleamine 2,3-dioxygenase (IDO) [12,13,14]. Metabolites of the kynurenine pathway play crucial roles in several physiological and pathophysiological processes [15,16,17,18,19,20,21,22,23,24]. Recent reports indicate that they are also connected with osteoblast proliferation and differentiation, and they can be related to the pathophysiology of osteoporosis [25,26]. Recently, we demonstrated that KYN produced in the central nervous system positively affects bone strength, while peripherally secreted KYN has the opposite effect [27,28].

The aim of this study was to establish whether the inhibition of 5-HT synthesis by LP533401 may modulate kynurenine pathway activity in bone tissue of nephrectomized rats and to determine the potential consequence of this process in relation to osteogenesis and regulation of bone mineral status.

## 2. Results

### 2.1. Effect of LP533401 Treatment on Tryptophan Utilization via the Kynurenine Pathway in Serum, Urine, and Intestinal Homogenate

**Figure 1:**
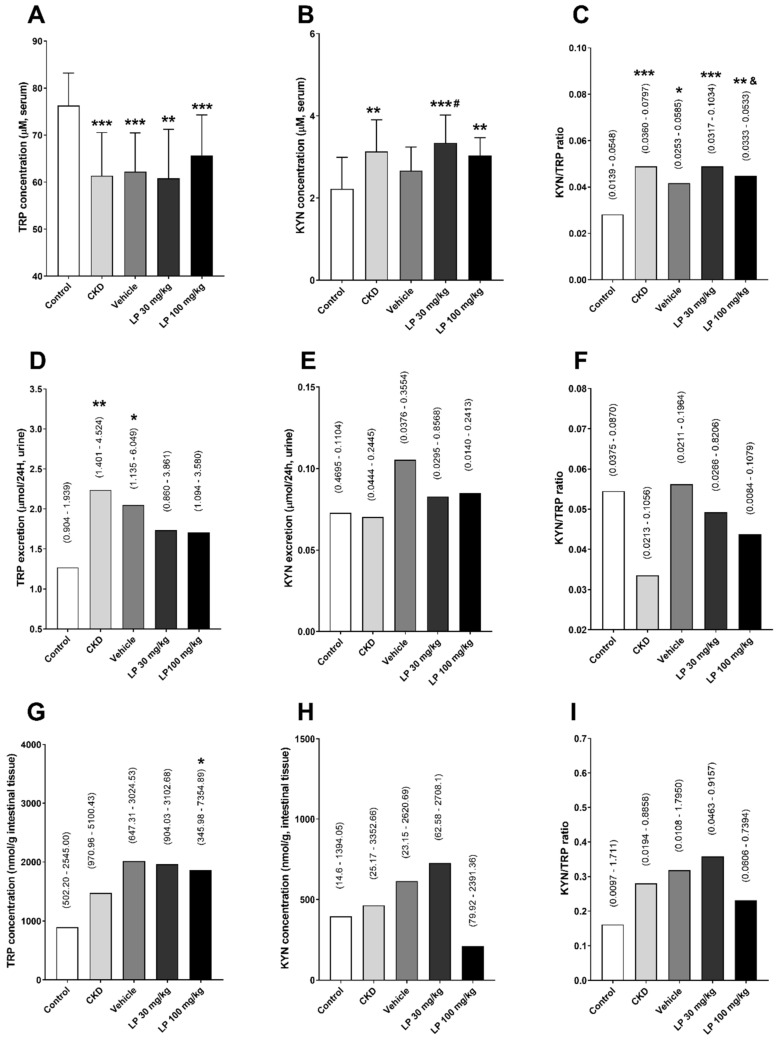
Changes in tryptophan (TRP) and kynurenine (KYN) concentrations, and KYN/TRP ratio among experimental groups in serum (**A**–**C**), diurnal urinary excretion (**D**–**F**), and intestinal homogenate (**G**–**I**). Data are shown as mean ± SD (**A**,**B**) or median and range (**C**–**I**). * *p* < 0.05, ** *p* < 0.01, *** *p* < 0.001, control vs. others; # *p* < 0.05, vehicle vs. others; & *p* < 0.05, LP 30 mg/kg vs. LP 100 mg/kg.

We observed that 5/6 nephrectomy caused a significant decrease in TRP concentrations and an increase in KYN levels and KYN/TRP ratio, being a marker of KYN pathway activation, in comparison with sham-operated animals. The administration of the LP533401 at the dose of 30 mg/kg (LP 30) caused a significant increase in serum KYN concentration, especially in relation to the vehicle group. We also found a significant decrease in the KYN/TRP ratio in the group treated with LP533401 at the dose of 100 mg/kg (LP 100) in comparison to LP 30 (Figure 1A–C).

As shown in Figure 1D–F, there were no differences in the diurnal KYN component excretion in urine, apart from increased TRP excretion in the CKD and vehicle groups compared to controls and LP533401-treated animals. Similarly, there were no differences in TRP, KYN levels, and the KYN/TRP ratio among all studied groups in the intestinal homogenate (Figure 1G–I).

### 2.2. Effect of LP533401 Treatment on KYN Pathway Activation in Bone Tissue

**Figure 2:**
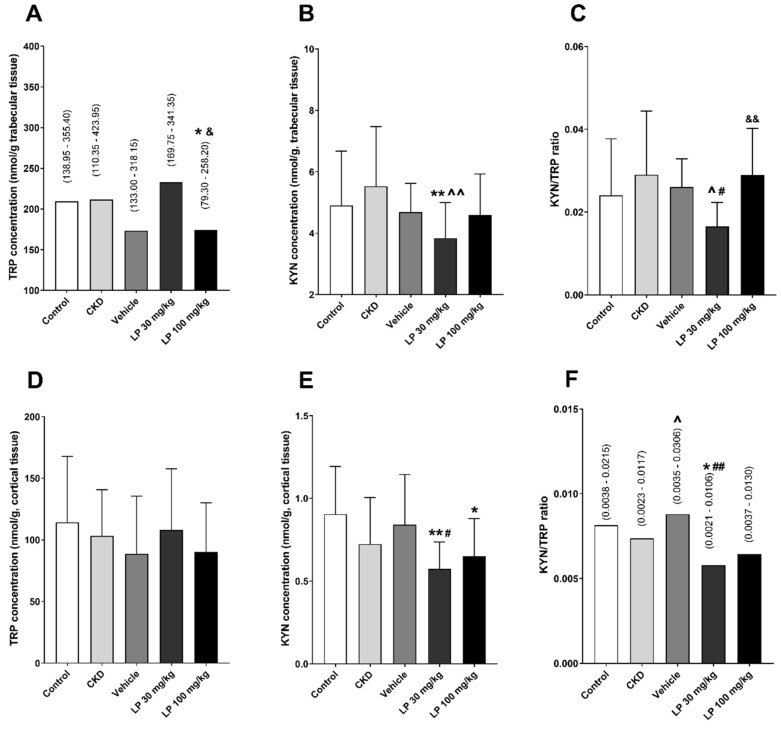
Changes of tryptophan (TRP) and kynurenine (KYN) concentrations, and KYN/TRP ratio among experimental groups in the femoral trabecular (**A**–**C**) and cortical (**D**–**F**) bone regions. Data are shown as median and range (**A**,**F**) or mean ± SD (**B**–**E**). * *p* < 0.05, ** *p* < 0.01, control vs. others; ^ *p* < 0.05, ^^ *p* < 0.01 CKD vs. others; # *p* < 0.05, ## *p* < 0.01, vehicle vs. others; & *p* < 0.05, && *p* < 0.01, LP 30 mg/kg vs. LP 100 mg/kg.

The concentrations of TRP and KYN, and the KYN/TRP ratio were measured separately in homogenates from the trabecular and cortical bone region. Lower TRP levels were observed in rats with CKD treated with LP 100 in comparison with the control (CON) and LP 30 groups. Despite this, the animals treated with LP at the dose of 30 mg/kg LP (LP 30) had lower KYN concentrations compared to CON and CKD in the trabecular bone. The higher dose of LP533401 resulted in an intensification of the KYN pathway activation in this bone region compared to other uremic groups, especially in relation to the LP 30 group (Figure 2A–C).

A similar tendency was shown in the cortical bone tissue homogenate. A significant decrease in KYN concentration was noticed in LP-treated groups compared to controls. We also found a significant increase in the KYN/TRP ratio after administration of the vehicle compared to the untreated CKD rats, as well as its decrease in the LP 30 group compared to the vehicle group and healthy controls (Figure 2D–F). Importantly, we did not observe an association between components of the KYN pathway in serum and bone tissue, which indicated an endogenous KYN synthesis in the bone.

**Figure 3:**
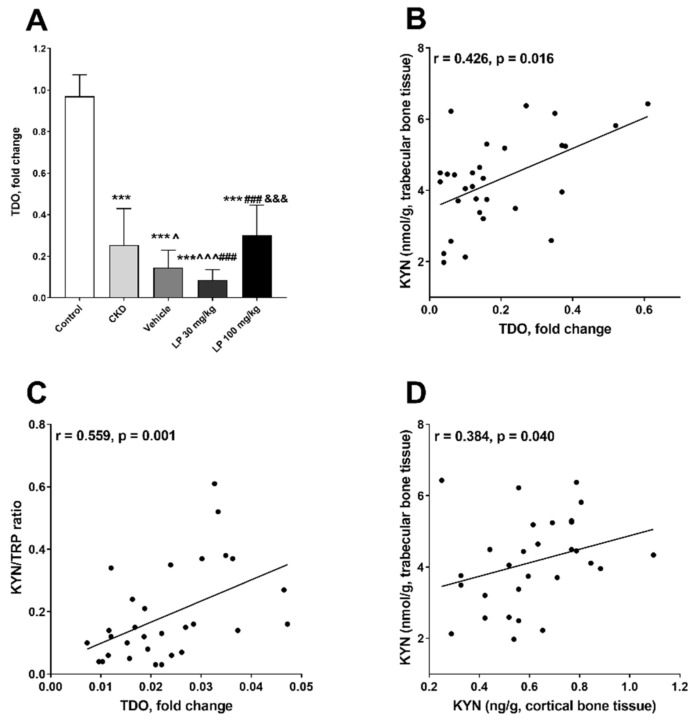
Changes in tryptophan 2,3-dioxygenase (TDO) expression level in the femoral bone (**A**) among experimental groups, and the association between TDO messenger RNA (mRNA) level and kynurenine pathway activation in bone tissue of CKD rats treated with LP533401 (**B**–**D**). Data are shown as mean ± SD. *** *p* < 0.001, control vs. others; ^ *p* <0.05, ^^^ *p* < 0.001, CKD vs. others; ### *p* < 0.001, vehicle vs. others; &&& *p* < 0.001, LP 30 mg/kg vs. LP 100 mg/kg.

Because KYN pathway activation can be IDO- or TDO-dependent [29,30], we tried to establish which of these enzymes may be responsible for KYN synthesis in the bone tissue, by measuring the expression of relevant genes. There was no IDO-1 and IDO-2 expression in bone tissue (data not shown), whereas TDO expression was present in all studied groups (Figure 3A). We observed a significant decrease in TDO messenger RNA (mRNA) level in the bone tissue of nephrectomized animals in comparison with the control. The administration of vehicle or LP 30 resulted in further attenuation of TDO expression in comparison with CKD. In the LP 100 group, the TDO mRNA level was still lower than in the control, but significantly higher than in the vehicle- and LP 30-treated groups (Figure 3A).

We also noticed that TDO expression was positively associated with KYN concentration and KYN/TRP ratio in the trabecular bone region (Figure 3B,C), and that KYN concentration in this bone region was positively related to KYN level in the cortical bone region (Figure 3D).

### 2.3. The Associations between Bone Mineral Status and Components of the Bone Kynurenine Pathway

**Figure 4:**
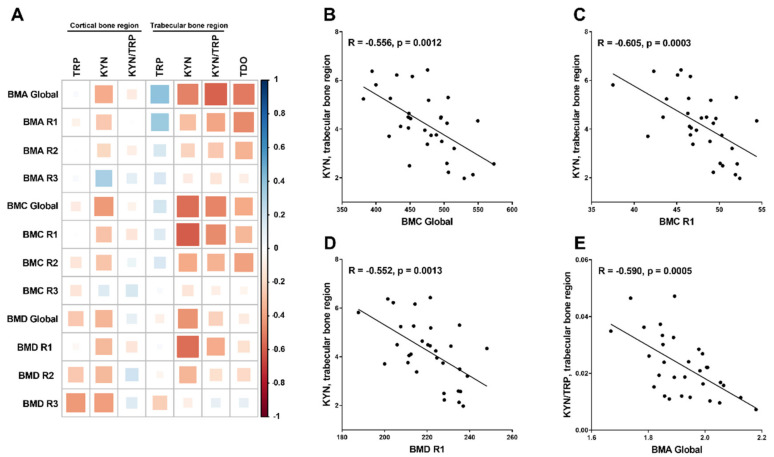
The association between components of the kynurenic system in bone homogenates and the parameters of bone mineral status in rats with CKD treated with LP533401 (**A**) and a detailed graphic presentation of the strongest among them (**B**–**E**). The size and the color intensification demonstrate the strength of the correlation (larger and darker circles represent a strong correlation). Blue colors—positive correlations; red colors—negative correlations. BMA—bone mineral area, BMC—bone mineral content, BMD—bone mineral density. R1—the distal metaphysis subregion, composed mostly of the trabecular tissue bone. R2—midshaft area subregion, constituted mostly of cortical bone tissue. R3—femoral neck subregion, built of a similar proportion of trabecular and cortical bone tissue.

The effect of LP533401 on femoral bone densitometry parameters was presented in detail in our previous study. Briefly, rats with CKD had significantly decreased bone mineral status compared with controls, while treatment with vehicle and LP533401 caused a significant increase in these parameters in comparison with untreated animals [11]. The evaluation of relationships between the components of the bone kynurenine pathway in individual bone regions and the bone mineral status in LP533401 treated rats revealed that TDO gene expression, as well as KYN concentration and KYN/TRP ratio in trabecular homogenates, was strongly and inversely related to bone mineral area (BMA), bone mineral content (BMC), and bone mineral density (BMD) values, particularly in the distal metaphysis (R1) bone region. In contrast, the TRP level in trabecular tissue was positively associated with BMA, especially in the R1 bone region. Similar, although weaker correlations were found between KYN levels in cortical homogenates and the studied parameters of femoral mineral status (Figure 4).

### 2.4. The Imbalance between Peripheral Serotonin and 25-Hydroxyvitamin D (25(OH)D) Status Affects TDO Expression in the Bone of CKD Rats Treated with LP533401

**Figure 5:**
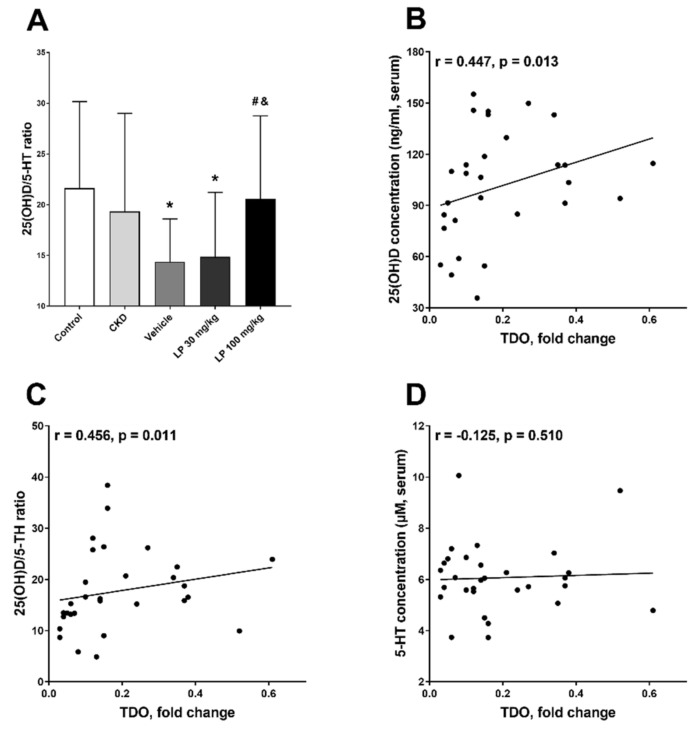
Changes in serum 25-hydroxyvitamin D to serotonin (25(OH)D/5-HT) ratio (**A**) among experimental groups, and the association between bone tryptophan 2,3-dioxygenase (TDO) expression and serum 25(OH)D concentration (**B**), 25(OH)D/5-HT ratio (**C**), and 5-HT level (**D**) in rats with CKD treated with LP533401. Data are shown as mean ± SD. * *p* < 0.05, control vs. others; # *p* < 0.05, vehicle vs. others; & *p* < 0.05, LP 30 mg/kg vs. LP 100 mg/kg.

In the next step of our work, we tried to find the factors that influenced bone TDO gene expression in CKD rats treated with LP533401. Previously, we showed that LP533401 administration to rats with CKD reduced turnover of circulating 5-HT [11], and it simultaneously caused the disturbances in serum 25-hydroxyvitamin D (25(OH)D) status [31]. In general, the dose-dependent rise in its concentration was observed in animals treated with LP533401 [31]. Recently, we demonstrated that not only peripheral 5-HT [32], but also the disturbances in calciotropic hormones, namely, the advantage of 25(OH)D over 5-HT, reflected by the 25(OH)D/5-HT ratio, can affect osteoblastogenesis in LP-treated animals. As shown in Figure 5A, 25(OH)D/5-HT ratio in the LP 100 group was comparable to the control, and it was significantly higher in this group than in the LP 30 and vehicle groups, where we observed a significant decrease in the 25(OH)D/5-HT ratio in comparison with the control. Moreover, both serum 25(OH)D levels, as well as 25(OH)D/5-HT ratio, were positively associated with TDO gene expression (Figure 5B,C), while serum 5-HT level did not correlate with TDO mRNA expression (Figure 5D).

### 2.5. The Association between Serotonin-Dependent Molecular Pathway Involved in Osteoblast Formation and Activity of Kynurenic System in the Bone of Rats with CKD Treated with LP533401

**Figure 6:**
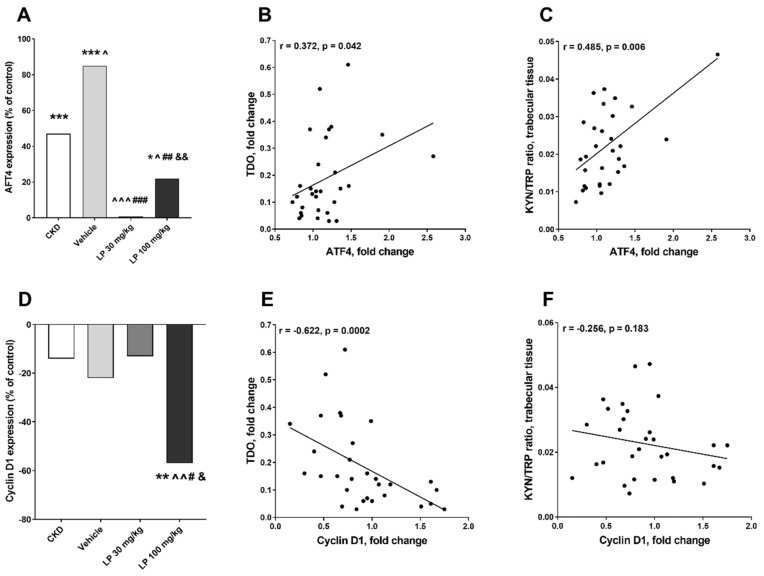
Changes in activating transcription factor (ATF4) and cyclin D1 gene expression among experimental groups (**A**,**D**), and their associations with tryptophan 2,3-dioxygenase (TDO) expression (**B**,**E**), and kynurenine to tryptophan (KYN/TRP) ratio in trabecular bone tissue (**C**,**F**) in rats with CKD treated with LP533401. Data relate to the control taken as 0%. * *p* < 0.05, ** *p* < 0.01, *** *p* < 0.001, control vs. others; ^ *p* < 0.05, ^^ *p* < 0.01, ^^^ *p* < 0.001, CKD vs. others; # *p*< 0.05, ## *p* < 0.01, ### *p* < 0.001, vehicle vs. others; & *p* < 0.05, && *p* < 0.01, LP 30 mg/kg vs. LP 100 mg/kg.

Data obtained from an in vitro experiment by El Refaey et al. suggest that KYN can impair osteoblastic differentiation from bone marrow mesenchymal stem cells and, via this mechanism, it can play a role in bone loss [33]. Herein, we analyzed the associations between the expression of 5-HT-dependent genes involved in osteoblast proliferation and differentiation processes [32] and the activity of the bone kynurenic system, represented as TDO expression and KYN/TRP ratio. As schematically presented in Figure 6A, activating transcription factor (ATF4) gene expression was similar in LP 30 and control groups, whereas it was significantly higher in other studied groups compared to healthy animals, and a positive relationship existed between ATF4 and TDO expression (Figure 6B), as well as between ATF4 gene expression and KYN/TRP ratio both in trabecular (Figure 6C) and in cortical bone tissue (*r* = 0.421, *p* = 0.023) of LP-treated rats. Moreover, a positive and strong association was also observed between ATF4 gene expression and trabecular KYN concentration (*r* = 0.474, *p* = 0.007). In contrast, cyclin D1 mRNA level was lowest in the LP 100 group compared to other studied groups (Figure 6D), and a strong inverse relationship existed between this gene’s expression and TDO expression (Figure 6E), whereas there was only a slight, non-significant relationship between cyclin D1 expression and the KYN pathway activation marker in rats with CKD treated with LP533401 (Figure 6F).

**Figure 7:**
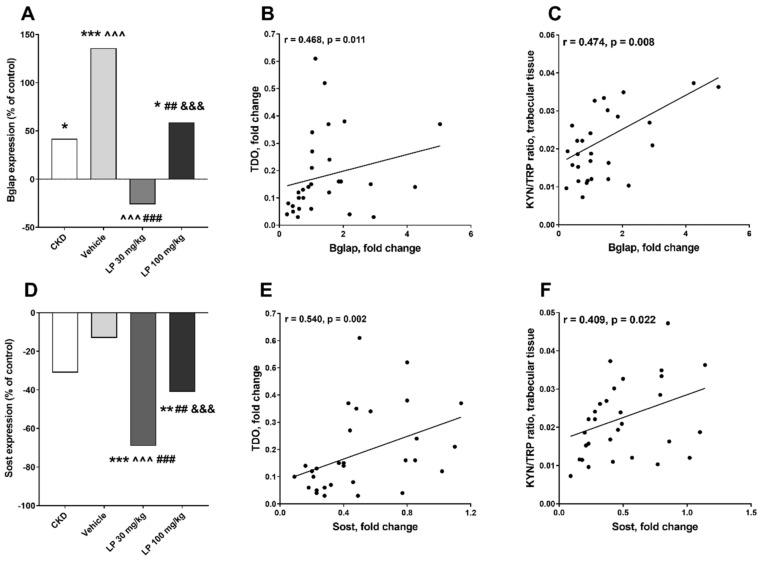
Changes in osteocalcin (Bglap) and (sclerostin) Sost gene expression among experimental groups (**A**,**D**), and their associations with tryptophan 2,3-dioxygenase (TDO) expression (**B**,**E**), and kynurenine to tryptophan (KYN/TRP) ratio in trabecular bone tissue (**C**,**F**) in rats with CKD treated with LP533401. Data relate to the control taken as 0%. * *p* < 0.05, ** *p* < 0.01, *** *p* < 0.001, control vs. others; ^^^ *p* < 0.001, CKD vs. others; ## *p* < 0.01, ### *p* < 0.001, vehicle vs. others; &&& *p* < 0.001, LP 30 mg/kg vs. LP 100 mg/kg.

Osteocalcin (Bglap) is a marker of the late stage of osteoblast differentiation, whereas sclerostin (Sost) is considered as an indicator of osteoblast transition to osteocyte [34]. As shown in Figure 7A, Bglap gene expression was higher in CKD and especially in the vehicle group compared to the control group. CKD rats treated with LP 30 had the lowest Bglap mRNA levels among all analyzed uremic groups, and a positive relationship existed between this gene’s expression and both TDO expression (Figure 7B) and KYN/TRP ratio in trabecular bone (Figure 7C) of uremic rats treated with LP533401. Similarly, Sost gene expression was significantly reduced in the LP 30 group in comparison with other studied groups (Figure 7D), and it was positively correlated with TDO expression (Figure 7E) and KYN/TRP ratio (Figure 7F) in trabecular bone tissue of rats treated with LP533401.

## 3. Discussion

Our study shows four main findings. Firstly, we established the presence of a TDO-dependent, paracrine kynurenic system in the bone of rats with CKD. Secondly, the modulation of this system during LP533401 treatment was associated with impaired bone mineral status. Thirdly, the imbalance between peripheral 5-HT and 25(OH)D affects TDO expression level and, consequently, KYN pathway activation in the bone of animals treated with LP533401. Fourthly, there are close associations between genes participating in osteoblastogenesis and TDO-dependent activation of the KYN pathway in the bone of uremic rats treated with LP533401.

The present study is a continuation and an extension of our earlier investigation concerning the impact of the inhibition of peripheral 5-HT synthesis by LP533401 on bone health, disturbance of calciotropic hormones, and the expression of 5-HT-dependent genes involved in osteoblastogenesis in uremic rats [11,31,32]. Previously, we also showed that the activation of the peripheral kynurenine system in young rats with mild to moderate CKD unfavorably affected bone microarchitecture geometry and strength [27]. In the present study, we measured the components of the KYN pathway, namely, TRP and KYN, and we determined KYN/TRP ratio, as a marker of this system’s activity in the blood, urine, intestinal, and bone tissue of rats with CKD treated with LP533401. The activation of the peripheral KYN system in uremic rats, observed in the present study, was in agreement with our previous experiments performed in the rat model of CKD [27,35,36], as well as with clinical data from CKD patients [37,38,39]. In the present study, we demonstrated for the first time the presence of TRP and KYN in bone tissue of all experimental groups. Next, we discovered that, among the three enzymes which can degrade TRP into KYN (IDO-1, IDO-2, and TDO), only TDO gene expression was present in bone, and it was associated with bone KYN levels and KYN/TRP ratio of studied animals. Moreover, there was no association between KYN levels in serum and bone tissue in these animals. The above results suggest that bone possesses its own, paracrine, TDO-dependent system, which is able to locally produce KYN [40]. Until now, the physiological constitutive expression of TDO was demonstrated only in liver and neurons [41]; thus, our unexpected finding pointing to TDO as an exclusive enzyme participating in TRP degradation to KYN in bone tissue may significantly expand the existing knowledge about peripheral TRP metabolism.

Despite significantly reduced TDO gene expression in rats with CKD and vehicle groups compared to the control, KYN concentrations and KYN/TRP ratio in bone tissue were comparable between these groups, suggesting the intensification of TRP degradation to KYN in uremic animals. The lowest activation of the bone kynurenic system observed in uremic rats treated with LP533401 at the dose of 30 mg/kg was compatible with the strongest reduction of TDO gene expression in these animals, compared to other groups. The pathophysiological consequence of the reduced activity of the KYN pathway in the bone of rats treated with LP533401 was an improvement of their mineral status, especially visible in the distal metaphysis (R1) bone region, which is rich in the more metabolically active trabecular bone. Previously, we showed that peripheral kynurenine levels correlated inversely with the parameters of bone biomechanics, bone geometry, and bone mineral status of young nephrectomized rats [27]. Taking these results together, the activation of both systemic and local KYN pathways in bone tissue seems to be related to an impairment in bone integrity in rats with CKD. This finding is in agreement with the observation made previously by El Refaey et al., where increasing the level of peripheral KYN resulted in accelerated skeletal aging and bone loss in mice [42]. In addition, a study reported an inverse connection between kynurenic acid, one of KYN metabolites, and BMD in humans [23]. The most likely explanation for this unfavorable effect of KYN on bone health may include the activation by KYN of the aryl hydrocarbon receptor-dependent pathway in the bone of CKD animals and humans [27,43]. This time, we also cannot rule out the presence of other pathological KYN interactions, leading to modulation of other signaling factors, such as the factor receptor activator of nuclear factor-κB ligand/osteoprotegerin axis and histone deacetylase-3 or runt-related transcription factor 2 expression [33,43].

**Figure 8:**
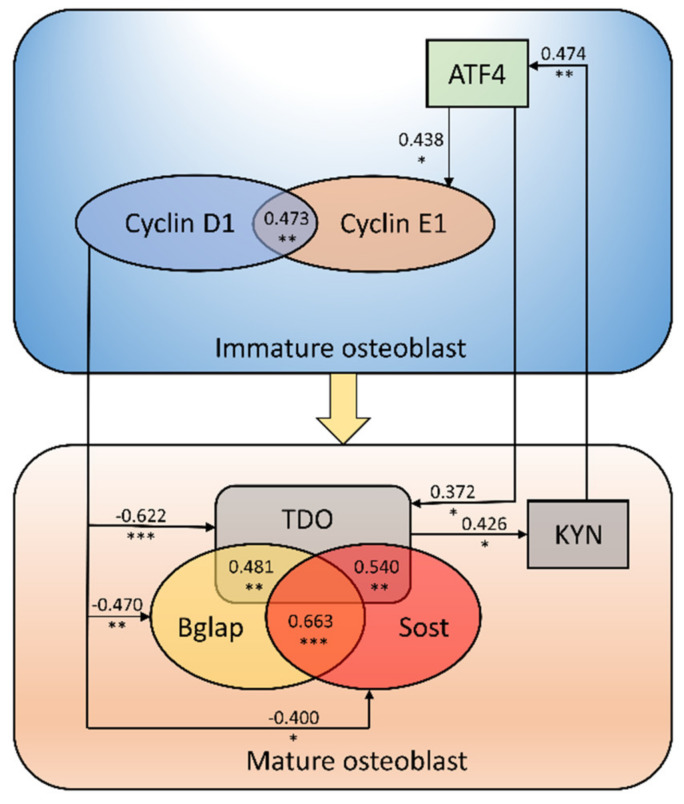
Schematic presentation of the possible role of bone kynurenic pathway activation in the osteoblast maturation process. Each value is Spearman’s rank correlation coefficient (*R*) value between the genes. * *p* < 0.05, ** *p* < 0.01, *** *p* < 0.001 indicate statistically significant values; a negative value indicates an inverse correlation between parameters. ATF4—activating transcription factor 4; Bglap—osteocalcin; KYN—kynurenine; Sost—sclerostin; TDO—tryptophan 2,3-dioxygenase.

In the next step of our study, we tried to identify the potential molecular mechanism leading to the increased TDO gene expression in the bone of CKD rats treated with LP533401 and its potential consequences. Previously, we noticed that the administration of LP533401 to rats with CKD, apart from the reduction of peripheral 5-HT, evoked disturbances in calciotropic hormones, resulting in an advantage of 25(OH)D over 5-HT [32]. Herein, we demonstrated that both serum 25(OH)D levels and 25(OH)D/5-HT ratio were positively associated with TDO gene expression. Thus, the imbalance between circulating 25(OH)D and 5-HT may be one of the factors affecting the activation of the bone KYN pathway during LP533401 treatment. Findings from our previous investigation also demonstrated that the imbalance between 5-HT and calciotropic hormones during LP533401 administration led to disruption of the interactions in the CREB–ATF4–FOXO-1 complex [32], which is is recognized as a crucial, 5-HT-dependent molecular pathway involved in bone formation [44]. The above mechanism resulted in the sequential and exclusive expression of genes involved in osteoblast growth, differentiation, maturation, and the improvement of bone mineral status in LP-treated animals [32]. The analysis of the interaction between genes involved in osteoblastogenesis and components of the bone KYN pathway revealed the association between the marker of the early stage of osteoblast proliferation/differentiation (ATF4) and the bone kynurenic system. On the other hand, stronger positive associations were noticed among TDO expression, KYN/TRP ratio, and the markers of terminally differentiated osteoblasts/osteocytes—Bglap and Sost gene expression (Figure 8). These results suggest that the activation of the kynurenic system is rather related to osteoblast maturation than to their differentiation process. The strong, inverse relationship between cyclin D1 (the key cell-cycle regulatory factor in proliferating/differentiating osteoblasts [45]) and TDO gene expression seems to confirm this hypothesis. Similar inverse associations were previously noticed by us between cyclin D1 and Bglap/Sost expression in this model [32]. In the available literature, there is scarce and contradictory evidence suggesting that degradation of TRP into KYN is essential for osteogenic differentiation. El Refaey et al. showed that KYN under in vitro conditions significantly inhibited bone marrow mesenchymal stem cell proliferation and differentiation into osteoblasts [33]. Opposing results were obtained by Vidal et al., who demonstrated that blocking the KYN pathway through IDO-1 inhibition led to impaired osteoblastic differentiation in vitro, and that IDO-1^−/−^ deficient mice were osteopenic [25].

It is worth noting that, in the present study, the components of the kynurenic system, especially in the trabecular bone region, were also positively related to ATF4 expression. Thus, it cannot be excluded that KYN may support the early stage of osteoblast differentiation, which is particularly intensified in CKD [32,46], while the inverse relationship between cyclin D1 and TDO gene expression may be an attempt to compensate for this phenomenon, in order to obtain mature osteoblasts (Figure 8) capable of performing their physiological functions, e.g., participation in the mineralization process. This is in line with the results of our previous study, in which the mitigation of intensified osteoblastogenesis in rats with CKD treated with LP533401 resulted in improvement of their bone mineral status [32].

In conclusion, our study for the first time demonstrated the presence of an active, paracrine kynurenic system in rat bone, which is independent of the peripheral one. The treatment of uremic rats with LP533401, which is an inhibitor of peripheral 5-HT synthesis, can indirectly activate this pathway, resulting in impaired bone mineral status. There are close associations among the expression of genes that participate in osteoblastogenesis, particularly with osteoblast maturation markers, and TDO-dependent activation of the KYN pathway in the bone of uremic rats treated with LP533401. Although it is not yet possible to fit all these data into a simple mechanistic hypothesis of the progression of osteoporosis in CKD, our results represent the next step in studying the role of tryptophan metabolites in renal osteodystrophy.

## 4. Materials and Methods

### 4.1. Animals and Experimental Design

Seventy-four male Wistar rats aged four weeks were purchased from and housed in the Centre of Experimental Medicine of the Medical University of Bialystok (Poland). The study was carried out in accordance with European Union (EU) Directive 2010/63/EU for animal experiments and was approved by the Local Ethical Committee on Animal Testing in Olsztyn (Permit Number: 29/2013, approved: 04/2013). The animals were housed in conventional cages, grouped as appropriate in vivarium conditions, with a 12-h light/dark cycle at controlled temperature (22 ± 2 °C) and humidity (55 ± 5%). They were allowed access to sterilized tap water and standard rat chow, containing 1% calcium, 0.7% phosphorus, and 19% protein. The animals’ health status was monitored throughout the experiments by a health surveillance program according to the Federation of European Laboratory Animal Science Associations (FELASA) guidelines. The rats and mice were free of all viral, bacterial, and parasitic pathogens listed in the FELASA recommendations. Ten of them were randomly chosen and sham-operated (control). The other 64 rats had induced CKD by surgical 5/6 subtotal nephrectomy using the surgical procedure described in detail previously [8]. Nephrectomized rats were randomized into four groups (*n* = 16 in each): untreated (CKD), administered with a vehicle, treated with LP533401 at a dose of 30 mg/kg for eight weeks, and treated with LP533401 at the dose of 100 mg/kg for eight weeks. After this period, the animals were subjected to analysis. The vehicle was polyethylene glycol and 5% dextrose in a ratio of 40:60. The doses of LP533401 were based on previous studies [10,47,48]. The vehicle or its solution with LP533401 was administered daily by gavage, while untreated nephrectomized and sham-operated rats received sterile water in this same regimen. At the end of the experiment, they were anesthetized until unconscious and euthanized by exsanguination via cardiac puncture. Procedures were conducted in the light phase of the cycle in the surgical room of our laboratory. Details about the experimental design, housing conditions, tissue collection, and general characteristics of animals were described in detail previously [11].

### 4.2. TRP and KYN Quantification in Serum, Urine, and Bone and Intestinal Tissues

Deproteinized serum samples were prepared by adding 50 µL of 2 M perchloric acid into 200 µL of a thawed sample. Then, 20 µL of urine samples were firstly diluted 10 times with distilled water and then acidified by adding 20 µL of 2 M perchloric acid. The acidified serum and urine samples were vortexed, kept at 4 °C for 20 min, and then centrifuged for 30 min at 14,000× *g* at 4 °C. The obtained supernatant was stored at −80 °C until assayed by high-performance liquid chromatography (HPLC, Agilent Technologies, Palo Alto, CA, USA).

Frozen small intestine samples were weighed and homogenized in 20% trichloroacetic acid in a ratio of 1:5. Obtained homogenates were placed at 4 °C for 30 min, and then the samples were centrifuged at 12,000× *g* for 20 min at 4 °C. After centrifugation, the supernatant was filtered (0.45-mm Millipore filter) and subjected to HPLC analysis.

Immediately after densitometric measurements, slices of bone tissue were taken from the distal femoral epiphysis (trabecular bone domination region) and femoral diaphysis (cortical bone domination region) subregions. Subsequently, they were weighed, thoroughly rinsed, and homogenized in a cold potassium phosphate buffer (50 mM, pH = 7.4; Polish Chemicals Reagents, Gliwice, Poland) using a high-performance homogenizer (Ultra-Turrax T25; IKA, Staufen, Germany) equipped with a stainless-steel dispersing element (S25N-8G; IKA, Staufen, Germany) to receive 10% homogenates. The homogenate was deproteinized by 20% trichloroacetic acid in a ratio of 1:4 and centrifuged at 14,000× *g* for 20 min at 4 °C; then, the supernatant was collected, filtered (0.45-mm Millipore filter), and stored at −80 °C. Immediately before performing the analysis, the supernatant was thawed and then injected into the HPLC system for the analysis.

The concentrations of TRP and KYN in serum, urine, intestinal homogenate, and trabecular and cortical bone tissue homogenates were determined using the HPLC method (Agilent 1260 series LC system, Agilent Technologies, Palo Alto, CA, USA) according to Holmes [49]. The prepared samples (2 µL) were separated on the ODS column (Waters Spherisorb 3 µm ODS2, 2.1 × 150 mm, Waters Corporation, Milford, MA, USA). The column effluent was monitored with a diode array detector (KYN-365 nm, TRP-260 nm, Agilent Technologies, Palo Alto, CA, USA). The mobile phase was composed of 0.1 M acetic acid and 0.1 M ammonium acetate (pH 4.6) containing 1.8% of acetonitrile, and it was pumped at a flow rate of 0.2 mL/min.

### 4.3. Tryptophan 2,3-Dioxygenase mRNA Expression Level Assessment

Total RNA was isolated from femoral tissue using a Thermo Scientific GeneJET RNA Purification Kit (Thermo Scientific, Vilnius, Lithuania), and a quantitative real-time polymerase chain reaction assay was performed, as described in detail previously [50]. Primers were designed using Primer-BLAST software. During the study, the expression of the TDO gene was assessed. The primer sequences were AGCGTCATGACTACCTTCTG and TGTCCATAAGTGAGGTCAGC(5′–3′ forward and reverse, respectively). All results were normalized to the endogenous reference glyceraldehyde 3-phosphate dehydrogenase. The comparative cycle threshold method was used for relative quantification of gene expression.

### 4.4. Statistical Analysis

Shapiro–Wilk’s test of normality was used for data distribution evaluation. Normally distributed data were expressed in the form of mean ± SD, while non-Gaussian data were shown as median and a full range. Multiple group comparisons were performed using the one-way analysis of variance (ANOVA), and significant differences between the groups were analyzed with the help of Duncan’s post hoc test at *p* < 0.05. The correlations between study variables were calculated with Spearman’s rank correlation analysis. A two-tailed *p*-value <0.05 was considered statistically significant. All computations for statistical analysis were performed using Statistica ver. 13.3 computer software (StatSoft, Tulsa, OK, USA). Graphic design presentation of the results was performed using R statistical software version 3.6.1 (The R Foundation for Statistical Computing, Vienna, Austria) or GraphPad Prism 6.0 software (GraphPad Software, San Diego, CA, USA).

## Abbreviations

25(OH)D	25-Hydroxyvitamin D
5-HT	Serotonin
ATF4	Activating transcription factor 4
Bglap	Osteocalcin
BMA	Bone mineral area
BMC	Bone mineral content
BMD	Bone mineral density
cAMP	Cyclic adenosine monophosphate
CKD	Chronic kidney disease
CREB	cAMP-responsive element-binding protein
FOXO1	Forkhead box protein O1
HPLC	High-performance liquid chromatography
IDO	Indoleamine 2,3-dioxygenase
KYN	Kynurenine
MBD	Mineral and bone disorders
RNA	Ribonucleic acid
Sost	Sclerostin
TDO	Tryptophan 2,3-dioxygenase
TPH	Tryptophan hydroxylase
TRP	Tryptophan
